# Klebsiella Pneumoniae Liver Abscess: a Case Report and Review of Literature

**DOI:** 10.7759/cureus.970

**Published:** 2017-01-10

**Authors:** Faisal Kamal, George Williams, Hina Akbar, Muhammad Ali Khan, Dipen Kadaria

**Affiliations:** 1 Medicine, University of Tennessee Health Science Center

**Keywords:** hepatic abscesses

## Abstract

Klebsiella pneumoniae (K.pneumoniae) is a known cause of pyogenic liver abscess (PLA) in the absence of hepatobiliary disease. In settings of hepatic infection, it has also been known to cause disseminated infections including meningitis and endopthalmitis. Several groups of patients are particularly susceptible to infection, including patients with diabetes mellitus, those from Southeast Asia and those with the preexisting hepatobiliary disease. We present a case of K.pneumoniae PLA with bacteremia. A 39-year-old Vietnamese male with no previous medical history who presented with complaints of abdominal pain, nausea, vomiting, diarrhea and fever. A computed tomography (CT) of the abdomen showed a large complex mass in the right lobe of the liver with multiple septations. Over course of hospitalization, the patient developed acute respiratory failure and was monitored in medical intensive care unit (MICU). Blood cultures grew K. pneumonia. The patient was treated with intravenous ceftriaxone and the abscess was drained by interventional radiology. After appropriate management, he progressed well during his hospital course and was eventually discharged from the hospital. K. pneumonia PLA had previously been an endemic disease in Southeast Asia, however, with a highly mobile patient population, it is now seen throughout the world and should be in the differential of patients who present with solitary liver mass in the setting of sepsis.

## Introduction

Klebsiella pneumoniae is a gram-negative organism that can cause pyogenic liver abscess (PLA) in the absence of hepatobiliary disease. Diabetics are at increased risk of this infection. Some patients with Klebsiella liver abscess can develop metastatic infections including endophthalmitis, meningitis, brain abscess, septic pulmonary emboli, lung abscess, splenic abscess, osteomyelitis, etc. It has been found to be a common cause of liver abscess in Taiwan. However, recently there has been the change in the trend of this infection in the United States [1]. And now it is one of the common causes of PLA in the United States. We present a case of Klebsiella liver abscess with bacteremia. Informed consent was obtained from the patient for this study.

## Case presentation

A 39-year-old Vietnamese male with no significant past medical history presented to a large urban emergency department with complaints of sharp 5/10 right upper quadrant abdominal pain, nausea, vomiting, fever and chills. The patient stated that he had been in his previous state of good health until approximately three days prior to presentation when his symptoms became debilitating. He worked at a local nail salon and stated that several co-workers were exhibiting cold like symptoms. His travel history was significant for the trip back to Vietnam within the last year but stated that his travels were uneventful and he did not remember feeling ill during that time. Review of systems was significant for the aforementioned symptoms in addition to loss of appetite with early satiety and one previous episode of streaky hemoptysis several days prior to presentation. In the emergency department, basic laboratory showed that he had an increased anion gap to 18, an acute kidney injury with a serum creatinine of 1.4, alkaline phosphatase 172, AST 81, ALT 107, ferritin 4,680 and a white blood cell count of 10.5.

A computed tomography (CT) scan with contrast of the chest and abdomen was ordered which was significant for a large 4.3 x 4.1 x 4.5 cm complex mass in the right lobe of the liver with multiple septations. Ultrasound of the abdomen showed a heterogeneous, hypoechoic, multiseptated hepatic mass with internal vascularity, however, a neoplastic, infectious or protozoa process could not be differentiated. He was admitted to the general medicine floor, was started on vancomycin and piperacillin/tazobactam with fluid resuscitation consisting of crystalloids, and blood cultures were ordered. Shortly after admission he became tachypneic, tachycardic and hypoxic with oxygen saturations in the 50s. At that time, he was placed on high-flow oxygen and had a rebound of his oxygen saturations to the low 90s, but still remained tachypneic and tachycardic. At this time, it was determined that he would benefit from admission to the medical intensive care unit (MICU). An arterial blood gas (ABG) was obtained and was significant for a pH of 7.34, a pCO2 of 26, and a bicarbonate of 14. He was placed on bilevel positive airway pressure ventilation and was continued on his antibiotics.

The repeat CT of thorax and abdomen was again significant for a large, complex hepatic mass, but now showed areas of consolidated infiltrate with some associated atelectatic change in the posterior right lower lobe. Magnetic resonance imaging (MRI) was recommended and showed a peripherally enhancing mass in the anterior segment of the right hepatic lobe that measured 3.5 cm x 3.3 cm with areas of central septations. Blood cultures grew Klebsiella pneumonia on the second day of admission with a MIC< 1 for ceftriaxone. He was placed on ceftriaxone and the abscess was drained by interventional radiology. The gram stain and cultures of pus were negative. The patient progressed well through his hospital course and was transferred out of the MICU after three days and from the hospital after 11 days. He was seen by infectious disease two months after discharge and a repeat CT of the abdomen showed interval improvement of the abscess.

## Discussion

Once thought to be isolated to Southeast Asia, pyogenic liver abscess (PLA) caused by Klebsiella pneumonia (K.pneumoniae), a potentially life-threatening cause of intra-abdominal infections in septic patients, is now seen across the World [2]. K.pneumoniae is now recognized as the most common isolate in both Asia and the USA and is seen in greater than 60% of monomicrobial and polymicrobial PLA infections [3-4]. The gram-negative K.pneumoniae is a well-known pathogen that is identified in multiple disease processes; however, what makes K.pneumoniae PLA unique is the patient population afflicted, its virulence and diagnostic features.

The most common presenting symptoms of K.pneumoniae PLA are fever, nausea, vomiting, and abdominal pain over the right upper quadrant. An elevated white blood cell count is almost universally seen. Asian ethnicity, recent antibiotic use, diabetes mellitus and impaired fasting blood glucose are the most important predisposing risk factors for developing K.pneumoniae PLA. Although males and those from Southeast Asia are over represented, PLA has been seen in all sexes and ethnicities. Diabetics tend to have monomicrobial abscess and disease process is typically more invasive with metastases to other organs, is often present and can lead to overwhelming sepsis. Septic endophthalmitis is strongly associated with the more virulent strains and diabetics [5]. Respiratory symptoms are often seen in invasive disease as the lungs are a common target for metastatic spread. The presence of meningitis, visual symptoms, dyspnea, cough, chest pain, large abscess size, all portend a worse prognosis and increased mortality [6].

Impaired neutrophil phagocytosis is what allows K.pneunoniae PLA to form in predisposed individuals. K1 K.pneumonia is the most commonly identified serotype seen in diabetics with invasive disease [5]. A novel gene, mapA, encoding for an outer membrane protein is commonly identified in invasive cases. Although, its exact virulence mechanism is not completely understood, when mapA is lost K.pneumoniae was susceptible to phagocytosis and became avirulent in mice. Because mapA is ubiquitous in virulent cases it could one day possibly be used as a laboratory marker for early identification in cases of suspected PLA. Hypermucoviscosity also plays a role in virulence in those with invasive disease, however, the exact mechanism of how hypermucoviscosity aides in virulence remain elusive.

Typical findings of K.pneumoniae PLA on abdominal computed tomography with contrast are a single, thin walled, multiseptated, solid masses with necrotic centers [7-8]. A predominately solid appearance is seen under ultrasound and aspiration often yields little pus with abundant necrotic material [9]. Monotherapy with an extended spectrum penicillins, such as piperacillin-tazobactam, or the third generation cephalosporin ceftriaxone in combination with metronidazole are first line therapies [10]. If unavailable, carbapenems or fluoroquinolones in conjunction with metronidazole can be used as second line therapy. Antibiotic resistance is more common in nosocomial infections; thankfully, however extended spectrum beta-lactamase strains (ESBL) are rarely seen. As with any abscess, prompt drainage with or without drain placement should be done early in the disease course.

## Conclusions

PLA should be part of the differential diagnosis of liver mass in the setting of sepsis, as K.pneumoniae is the most common isolate from both monomicrobial and polymicrobial PLA. Diabetes mellitus is the single most important risk factor for contracting K.pneumoniae PLA. Imaging of K.pneumoniae PLA often shows a solid, multiloculated mass. K.pneumoniae PLA, in particular, has a propensity to metastasize to other organs and cause systemic symptoms, and prompt drainage along with antibiotics drastically decrease morbidity and mortality.
